# The Plasticity of Root Systems in Response to External Phosphate

**DOI:** 10.3390/ijms21175955

**Published:** 2020-08-19

**Authors:** Guoqiang Huang, Dabing Zhang

**Affiliations:** 1 Joint International Research Laboratory of Metabolic & Developmental Sciences, SJTU-University of Adelaide Joint Centre for Agriculture and Health, School of Life Sciences and Biotechnology, Shanghai Jiao Tong University, Shanghai 200240, China; huang19880901@126.com; 2 School of Agriculture, University of Adelaide-SJTU Joint Centre for Agriculture and Health, Food and Wine, University of Adelaide, Waite Campus, Urrbrae 5064, South Australia

**Keywords:** phosphate, root system architecture, *Arabidopsis*, rice

## Abstract

Phosphate is an essential macro-element for plant growth accumulated in the topsoil. The improvement of phosphate uptake efficiency via manually manipulating root system architecture is of vital agronomic importance. This review discusses the molecular mechanisms of root patterning in response to external phosphate availability, which could be applied on the alleviation of phosphate-starvation stress. During the long time evolution, plants have formed sophisticated mechanisms to adapt to environmental phosphate conditions. In terms of root systems, plants would adjust their root system architecture via the regulation of the length of primary root, the length/density of lateral root and root hair and crown root growth angle to cope with different phosphate conditions. Finally, plants develop shallow or deep root system in low or high phosphate conditions, respectively. The plasticity of root system architecture responds to the local phosphate concentrations and this response was regulated by actin filaments, post-translational modification and phytohormones such as auxin, ethylene and cytokinin. This review summarizes the recent progress of adaptive response to external phosphate with focus on integrated physiological, cellular and molecular signaling transduction in rice and *Arabidopsis*.

## 1. Introduction

Phosphate is an macroelement required for almost all biologic activities of plants, such as photosynthesis, nitrogen fixation, carbohydrate metabolism, energy generation, nucleic acid synthesis, enzyme activation/inactivation, signaling transduction [1]. Phosphate starvation causes extensive losses to agricultural production by impairing plant growth and reducing yield [2]. By estimation, more than 30% world’s arable land has insufficient available phosphate required for crop growth [2]. At the current rate of consumption, the finite and non-renewable natural resources of inexpensive phosphate may be depleted before 2050 [2,3,4,5]. Nevertheless, the efficiency of phosphate uptake by crop root system is still below 25% due to the accumulation of phosphate at the topsoil in many natural and agricultural ecosystems [5,6]. In addition, excessive phosphate has been applied for the arable farming system and then discharged into the environment, resulting in severe water pollution [7]. Therefore, the improvement of phosphate uptake efficiency is critical for the reduction of fertilizer input, achieving the environmentally sustainable agriculture [8].

**Figure 1:**
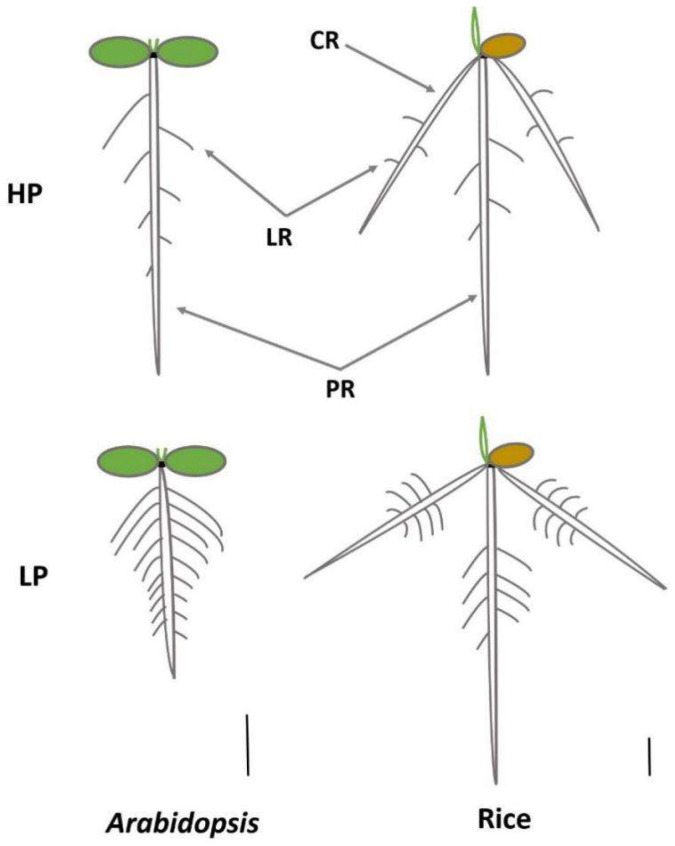
Schematic representation of a typical allorhizic (left) and secondary homorhizi (right) root system grown in HP (upper) and LP (lower), exemplified by *Arabidopsis* and rice. Shallow and deep root systems were formed in LP or HP, respectively. Shorter PR length, longer length and higher density of LR and root hair were formed for *Arabidopsis* in LP; Larger CR growth angle and longer length and higher density of root hair were developed for rice in LP. PR—primary root; LR—lateral root; CR—crown root; HP—high phosphate conditions; LP—low phosphate conditions. Scale bars, 1 cm.

On the other hand, to overcome phosphate deficiency, plants have evolved various adaptive mechanisms to improve the efficiency of phosphate uptake [9]. Root system architecture is highly plastic in response to various phosphate conditions by modulating the length of primary root and length/density of lateral root, root hair and crown root angle [8,10,11,12,13,14,15]. Dicot plants such as *Arabidopsis thaliana* has the tap root system with the dominant primary root and branches consisting of secondary, smaller lateral roots and root hairs [15] (Figure 1). While monocot plants, such as rice (*Oryza sativa*), wheat (*Triticum aestivum*) and maize (*Zea mays*) develop fibrous root systems mainly consisting of adventitious roots (or called crown roots in cereals) [15] (Figure 1). For rice, adventitious roots are mainly formed from postembryonic shoot-borne roots, and primary and crown roots have the capacity to branch by forming lateral roots and generating root hairs from their epidermal cells. Given that different root system morphologies were gradually formed during the evolution for the two species. *Arabidopsis* and rice have developed distinct mechanisms to enhance topsoil-exploring abilities under low phosphate conditions. Shallow root system was formed through reducing primary-root length, increasing root hair/lateral root density/length in *Arabidopsis* under low phosphate conditions. Unlike that of *Arabidopsis*, rice would develop this system mainly via increasing crown-root growth angle and root hair length to cope with low phosphate conditions (Figure 1).

Researchers have created artificial system with adding or reducing the exogenous phosphate (without changing chloride concentration) to mimick the high or low phosphate conditions (rather than chloride toxicity) in the natural environments. Through genetic screening approaches, they have identified a series of regulators modulating root system architecture in response to external phosphate, and these factors have formed a regulatory cascade described in previous review articles [8,11,12,13,14]. In this review, we focus on the recent understanding in the interplay between phosphate signaling pathways and plant hormones, actin filaments and post-translational modifications in regulating root system architecture of *Arabidopsis* and rice.

## 2. Root System Architecture Modulated by Phytohormones in Response to External Phosphate

### 2.1. Auxin

It is well known that auxin regulates root patterning in response to phosphate starvation [8,11,13,14]. Exogenous auxin treatment caused the localized architecture alteration of phosphate-adequate roots, mimicking that of roots cultured under low phosphate conditions [16]. Similar to the response of low-phosphate grown root system, the treatment of exogenous auxin caused the increase of *Arabidopsis* lateral-root density which depends on the auxin receptor TRANSPORT INHIBITOR RESPONSE1 (AtTIR1) and two auxin response factors, AUXIN RESPONSE FACTOR7 (AtARF7) and AtARF19 [17,18]. Furthermore, the promotor of *PHOSPHATE STARVATION RESPONSE 1* (*AtPHR1*) which encodes one MYB transcription factor with the induced expression by phosphate starvation, contains three auxin-response elements that can be directly bound by AtARF7 and AtARF19 to promote its expression under low phosphate conditions [14]. AtPHR1 and OsPHR2 (the rice homolog of *Arabidopsis* PHR1) play a conserved and central roles in phosphate utilization by regulating phosphate signaling and homeostasis to assist plant adaption to phosphate deficiency by binding to the *cis*-element with an imperfect palindromic sequence (GnATATnC, i.e., PHR1 binding site [P1BS]) [19,20,21,22,23]. The promotors of *PHOSPHATE TRANSPORTER 1* (*AtPT1*) and *OsPT2* encoding phosphate transporters contain P1BS motifs, which can be physically bound by AtPHR1 and OsPHR2, respectively [21,24]. There are 9 and 13 high-affinity PTs in *Arabidopsis* and rice and accounting for about 70% of the total root phosphate transport activity [25,26]. Besides the *PTs*, *INDUCED BY PHOSPHATE STARVATION1* (*IPS1*), microRNA399 (miR399) and some phosphate signaling components were also directly regulated by PHR1/OsPHR2 in phosphate uptake, translocation and response [19,20]. A systemic framework has been proposed based on the above phosphate-signaling components, which were downstream regulators of AtARF7/19. Therefore, the changes of lateral root density under low phosphate is in an auxin-dependent manner.

**Figure 2:**
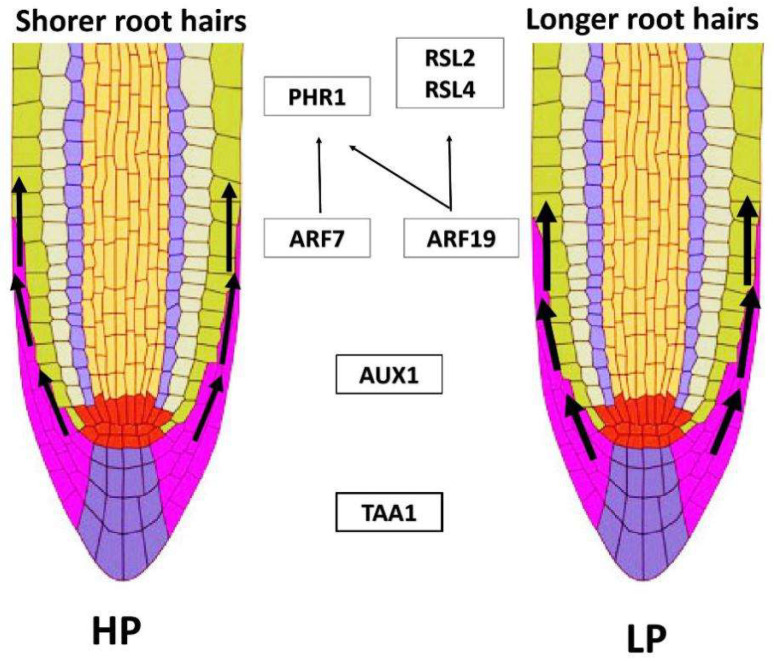
Schematic illustration of root-hair elongation under low phosphate conditions [13,14]. LP elevated the auxin levels of elongation and root-hairs zone by increasing auxin biosynthesis from higher expression of *AtTAA1* in root tip and enhanced AUX1-mediated auxin transport through lateral root cap and epidermal cells. The increased auxin levels lead to the induced expression of *AtRSL2*/*4* and *AtPHR1* regulated by AtARF7/19 in elongation zone and differentiation zone, finally promoting root-hair elongation. Black arrows indicate the auxin transport rate and direction (left, less auxin transport; right, more auxin transport). HP/LP—high/low phosphate conditions.

Unlike the increasing in density of lateral root under phosphate starvation conditions, the auxin-induced root hair growth under this condition is evolutionarily conserved in *Arabidopsis* and rice [8,13]. Exogenous auxin can increase the length of root hair under high phosphate conditions, but not under low phosphate conditions in both rice and *Arabidopsis* [8,13]. Without exogenous auxin, *aux1* (an auxin influx carrier mutant) showed reduced phosphate uptake and arrested root hair expansion in low phosphate conditions [13]. Targeted expression of *AtAUX1* in lateral root cap and epidermal cells can restore root-hair defects under low phosphate [13], demonstrating that low phosphate-induced root-hair elongation requires the mobilization of auxin from root apex to root-hair zone via AUX1 (Figure 2). There are five closely related *OsAUX1*/*LAX* genes in rice genome [8]. Among them, only *OsAUX1,* but not others can complement the defective gravity response of *aux1–22* in *Arabidopsis*, indicating that AUX1 performs a conserved role in the two species [8]. Intriguingly, *osaux1* mutants exhibited shallow root system, but reduced phosphate uptake efficiency owing to shorter root-hair length caused by less auxin response of root epidermal cells under low phosphate conditions [8]. Auxin response in epidermis is elevated as a result of the increased auxin biosynthesis at the root apex under the low external phosphate conditions, and then auxin was mobilized to the root-hair zone in an OsAUX1-dependent manner [8], which is also conserved in *Arabidopsis*. Moreover, the elevated level of auxin at elongation zone and root hair zone was shown to be caused by the increased expression level of auxin biosynthesis gene *TRYPTOPHAN AMINOTRANSFERASE OF ARABIDOPSIS1* (*AtTAA1*) in the root apex and AUX1-mediated auxin transport [13]. In addition, *ROOT HAIR DEFECTIVE 6-LIKE 2* (*AtRSL2*) and *AtRSL4* were upregulated with redundant roles in promoting root-hair elongation when exposed to low phosphate conditions [13]. Therefore, we propose a mechanistic regulatory framework of root-hair elongation mediated by auxin under phosphate-starvation conditions (Figure 2).

### 2.2. Ethylene

Several studies have suggested that ethylene functions in modulating root architecture in low phosphate conditions [27,28,29]. The transcript levels of ethylene biosynthesis genes were upregulated in low phosphate conditions [30]. Moreover, the addition of ethylene precursor 1-aminocyclopropane-1-carboxylic acid (ACC) inhibited primary root growth and lateral root formation in both low- and high-phosphate environments [17]. Yet, the precise roles of ethylene in regulating root adaptions to phosphate availability are still unclear. It appears that ethylene regulates low phosphate-induced root response via changing its biosynthesis and responsiveness [31]. The *ETHYLENE OVERPRODUCER 1* (*AtETO1*) and *CONSTITUTIVE TRIPLE RESPONSE 1* (*AtCTR1*) mutants showed less response to phosphate-starvation conditions [17]. Therefore, both the biosynthesis and responsiveness of ethylene were demonstrated to modulate primary root elongation and lateral root formation in response to external phosphate availability.

The initiation and elongation of root hairs were induced when exposed to more ethylene produced in low phosphate condition [27]. The low phosphate-induced root-hair growth results from the increased number of hair cells and shortening trichoblast cells [27], whilst this phenotype was strongly suppressed in ethylene-insensitive mutants, such as *ETHYLENE INSENSITIVE 7* (*atein7*) and *ETHYLENE INSENSITIVE ROOT 1* (*ateir1*/*pin2*) [27]. Moreover, several studies reported that both low phosphate conditions and ethylene regulate root elongation via stimulating auxin biosynthesis of root apex and facilitating AUX1-mediated auxin transport along lateral root cap and epidermal cells of meristem and elongation zone [13,32,33,34]. In addition, OsYUCCA8 (OsYUC8)-mediated auxin biosynthesis genetically acts downstream of OsEIN3-LIKE1 (OsEIL1), which are sufficient for activating the expression of ethylene-response genes required for root phosphate-starvation response [35]. Hence, ethylene is likely utilized to fine-tune the low phosphate-induced adaptations of root system in an auxin-dependent manner.

### 2.3. Cytokinin

Cytokinin is proved to play positive and negative roles in shoot and root growth, respectively [36]. The activity of root apical meristem was inhibited by exogenous cytokinin, causing a reduction of low-phosphate response [37]. Moreover, the expression of *INDUCED BY PHOSPHATE STARVATION 1* (*AtIPS1*) and other phosphate-starvation response genes were repressed with the treatment of cytokinin in *Arabidopsis* [38]. In turn, low phosphate represses cytokinin responsiveness and sensitivity through reducing its concentrations [39] and decreasing the expression of *CYTOKININ RESPONSE 1* (*AtCRE1*) (a cytokinin receptor) [40]. Thus, an antagonistic relationship was believed to exist between cytokinin and phosphate starvation signaling.

However, the changes of cytokinin signaling triggered by low phosphate are the second response, as a consequence of “crosstalk” between auxin and phosphate signaling cascades [41]. It is reported that cytokinin biosynthesis via isopentenyladenosine-5′-monophosphate-independent pathway was suppressed by auxin in a fast way [41]. In turn, cytokinin perturbs the expression of auxin efflux carriers *PINs* and disturbs the pattern of lateral root primordia via preventing the formation of auxin gradient [42]. These findings demonstrate that auxin and cytokinin play opposite effects in lateral root formation induced by low phosphate conditions.

Besides the above phytohormones, brassinosteroid (BR) was also proved to regulate root morphology in low phosphate conditions [43]. Transcription factor BRI1-EMS-SUPPRESSOR 1 (AtBES1) in BR signaling pathways is essential for maintaining normal root morphology under phosphate starvation conditions [43]. Despite that some functions of phytohormones in low phosphate response have been uncovered, and more potential roles await to be revealed.

## 3. The Roles of Actin Filaments in Crown-Root Angle Adjustment

**Figure 3:**
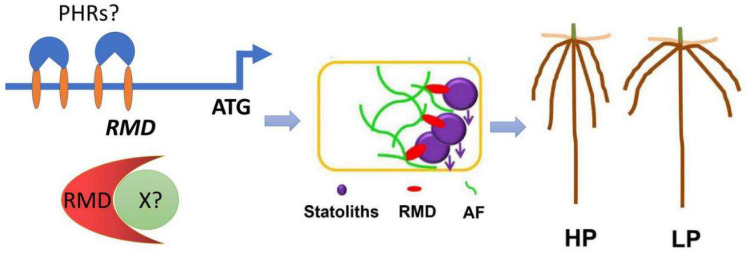
Proposed model for phosphate-dependent RMD-mediated regulation of RSA [12]. RMD links statoliths and actin filaments (AFs) in root columella cells, which is essential for RSA adaptation to different phosphorus conditions through fine-tuning gravitropism. RMD links statoliths and AFs in root columella cells which is essential for RSA adaptation to different phosphate conditions through fine-tuning gravitropism. There are 4 PHR1-like motifs among *RMD* promotor and which likely to be physically bound and activated by PHRs in rice. RMD-interacting proteins were necessary to elucidate the underlying mechanism in this response. In HP, the expression of *RMD* is lower, gravity-sensing process is enhanced and deep root system was shaped. In LP, the expression of *RMD* is higher, gravity-sensing process is dampened and shallow root system was developed.

Crown roots are major components of rice root system and their growth angle determines the phosphate uptake efficiency [44]. To survive in many natural and agricultural ecosystems with limited phosphate resource in the topsoil [45,46], crops such as rice has to increase phosphate uptake efficiency at minimum cost through increasing their crown-root growth angle to enlarge the exploring area within the topsoil [47,48]. Rice MORPHOLOGY DETERMINANT (RMD) is a type II formin homology 5 (OsFH5) and regulates the dynamics of actin filaments [49]. RMD is localized on the outer membrane of chloroplast or statolith within leaf epidermal or root columella cells, and acts as a scaffold protein to directly link actin filaments and chloroplasts/statoliths, respectively [11,50] (Figure 3). Loss-of-function mutants of *RMD* exhibited an enhanced gravitropic response due to rapider statoliths movement in root tip [11]. Moreover, the expression level of *RMD* was negatively correlated with the external phosphate availability, and RMD is key to the transition between deep and shallow root systems under high- and low-phosphate conditions, respectively (Figure 3) [11]. In high phosphate conditions, the transcript level of *RMD* was downregulated, causing less ring-like actin filaments surrounding statoliths, quicker movement of statoliths and enhanced gravity-sensing process and finally forming deep root systems (Figure 3) [11]. On the contrary, low-phosphate conditions induced the expression of *RMD* and more ring-like actin filaments around statoliths slow down the sedimentation of statoliths and gravity-sensing process, ultimately causing shallow root system (Figure 3) [11]. Therefore, RMD appears to function in buffering gravity-sensing process responding to external phosphate levels, which provides new mechanistic insights into how plants regulate key components of gravitropic machinery to adapt their root-systems architecture to soil phosphate availability (Figure 3) [11]. Meanwhile, the extent to which upregulation of *RMD* at the transcript level is responsible for this action needs more investigation [11]. For instance, the transcription factors that modulate the expression level of *RMD* remain to be elucidated to clarify the molecular mechanisms of adaptive response to external phosphate (Figure 3). Moreover, RMD acts as a scaffold protein and its interactors are vital to uncover the regulatory mechanisms of RMD at the protein level (Figure 3). In addition, there are another 15 formin members in rice genome and their roles in the adaptive response to other environmental cues are fascinating and remain to be investigated in future.

## 4. Post-Translational Modification in Phosphate Signaling

As a more precise mechanism, post-translational regulation such as ubiquitination and SUMOylation (small ubiquitin-like modifier peptide is attached to protein) in phosphate starvation response has been well studied [51,52]. SUMOylation alters protein activity, subcellular localization or the ability of interaction instead of triggering their targets degradation through 26S proteasome or vacuole, conventionally discovered in ubiquitination [53]. Here we briefly summarize the mechanisms of the two post-translational modifications in affecting phosphate uptake, long-distance phosphate signaling and phosphate starvation responses.

### 4.1. Ubiquitination

**Figure 4:**
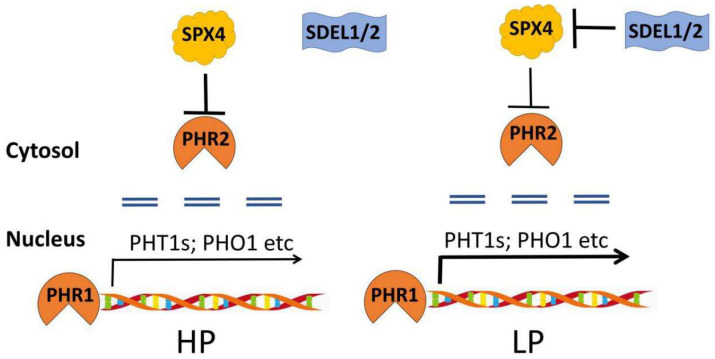
Ubiquitination-mediated translational regulation of the phosphate starvation response [60,61]. In high phosphate conditions, OsSPX4 interacts with OsPHR2, and reduces its ability to bind P1BS motifs and activate the expression of downstream regulators. In low phosphate conditions, OsSDEL1/2 competes with OsPHR2 to interact with OsSPX4, resulting in the degradation of OsSPX4 and the liberation of OsPHR2. OsPHR2 is translocated into the nucleus, which activates the expression of the downstream genes. Double horizontal dotted lines mean the nuclear membrane. The thickness of black arrows indicate the transcriptional activation level.

Ubiquitin (Ub) is a small peptide and covalently linked to lysine residues of targeted proteins through specific enzymatic cascades, which began by transferring a Ub moiety from an E1 Ub-activating enzyme (E1) to an E2 Ub-conjugating enzyme (E2). Then, E3 Ub-ligases (E3) brings together the E2 and the targets which were then ubiquitinated [54]. After several cycles of ubiquitination, the targets were polyubiquitinated and subsequently recognized by the 26S proteasome or vacuole for degradation, which depends on the way of ubiquitin linkage [54]. Low phosphate conditions accelerate the degradation of AUXIN/INDOLE-3-ACETIC ACID (AtAUX/IAA), thereby unshackling AtARF19 that activates the expression of *AtPHR1* [14,18]. Phosphate-starvation mediated root-system-architecture remodeling partially depends on the AtPIN2 accumulation through lysine^63^-linked plasma membrane (PM) cargo ubiquitylation [55,56]. Moreover, active PTs were relocated on the PM to increase phosphate uptake, by contrast, the PM-accumulated PTs were degraded in phosphate-sufficient conditions [23,57]. The proteins of AtPT1–4 were accumulated in the knockout mutants of *PHOPSHATE 2* (*AtPHO2*), which encodes an endosome-localized E2 ubiquitin-conjugating enzyme [58]. The RING-type E3 ligase NITROGEN LIMITATION ADAPTATION 1 (NLA1) was reported to interact with PTs and target their degradation and *nla1* has a higher phosphate contents conserved in *Arabidopsis* and rice [23,59]. In addition, the transcriptional activities of PHRs are negatively regulated solely by SYG/Pho81/XPR1 (SPX) domain-containing proteins (SPXs), which could interact with PHRs and inhibit their binding ability to P1BS motifs under high phosphate conditions that is conserved in *Arabidopsis* and rice [60,61]. However, the RING-type E3 ligases OsSDEL1 and OsSDEL2 (SPX4 degradation E3 ligases 1 and 2) interact with OsSPX4, causing its degradation and the liberation of OsPHR2 in phosphate starvation conditions [60,61] (Figure 4). The ubiquitination-mediated regulation of SPX4 deepens our understanding on the phosphate signaling cascade.

### 4.2. SUMOylation

SUMO is an ~100-amino-acid polypeptide and SUMOylation is a reversible process erased by deSUMOylating proteases (DSPs) that specifically cleave the isopeptide linkage between SUMO and the targets [62]. Recent studies demonstrated that plant SUMOylation plays vital roles in phosphate starvation response [63]. The SUMO E3 ligase SAP and Miz1 (AtSIZ1) regulates root patterning under phosphate starvation [63] and *siz1* exhibits reduced length of primary root and increased lateral root proliferation in phosphate-insufficient conditions [63]. Low Phosphate Root1 (AtLPR1) and AtLPR2 were respectively SUMOylated by AtSIZ1 and another SUMO ligase METHYL METHANE SULFONATE SENSITIVITY 21 (AtMMS21), which regulate local phosphate sensing in phosphate-starvation response [64,65]. Purple acid phosphatase 10 (AtPAP10) and AtPAP26 were responsible for internal phosphate recycling or releasing phosphate from external organophosphates for plant uptake in phosphate-deprivation conditions [66,67]. AtPAP10/26, AtARF7 and AtPHR1 could also be SUMOylated [63,66,67], while whether these processes affect their function in response to phosphate starvation requires further study. As an emerging post-translational modification in plants, more SUMOylation targets and their roles need to be identified in phosphate signaling pathway.

Overall, recent studies have addressed the significant roles of post-translational modifications, which regulate phosphate-starvation response at morphologic, physiological, biochemical and molecular levels. Despite some progresses have been made, many interesting questions remain to be answered on how plants employ post-translational modification for a higher phosphate-uptake efficiency.

## 5. Conclusions and Future Perspectives

Based on previous studies and the above discussion, the molecular mechanisms of the adaptive response of root system to external phosphate has been well described [11,12,13]. Low phosphate availability alters the local concentrations, transport or sensitivity of intracellular components and subsequently modulates the expression level of the downstream genes that control the initiation, adjustment and maintenance of root system development. Some progresses have been achieved to open inroads to understand the mechanisms of phosphate sensing and response. Root cap was demonstrated to perform the roles to sense and/or response to low concentrations of environmental phosphate by split root experiments in *Arabidopsis* [13,50]. Therefore, the functional analysis of the different root tissues of the root tip will be crucial to identify the early steps of these process. In addition, how external phosphate is sensed by plants is always a fascinating subject that deserved to be more investigation.

As a macro-element, phosphate is easily accumulated in the topsoil [1]. Regarding this, shallow root system have advantages in absorbing limited phosphate from the topsoil [11]. Previous works were mainly focus on the local architectures of root system, such as primary root length, the length/density of lateral root/root hair and crown root growth angle [10,11,12,13,14,15]. However, the formation of shallow root system requires coordinated activities of different-type roots. For *Arabidopsis*, many studies have uncovered that the regulatory mechanism of root system in response to external phosphate. Compared with that of *Arabidopsis*, the reported studies were mainly focus on the length of primary/crown root in rice [26,59,60,61]. Crown root growth angle as an agronomic architecture determining the transition between shallow and deep root system was less investigated. RMD is type II formin (FH5), regulates the dynamics of actin filaments and involves the transition between shallow and deep root systems [11]. The post-transcriptional and post-translational modification of RMD, which is vital to uncover its regulatory mechanisms, should be elucidated in further work. Moreover, RMD-like proteins that regulate root system architecture responding to external phosphate are worth more attention. In addition, a perfect shallow root system (not local changes) is necessary to help plants perform better (especially high yield) in low phosphate conditions, and reduce the use of phosphate fertilization, and promote the development of sustainable agriculture.

Phosphate shortage is becoming severely urgent associated with the increasing demands created by human activities [3,5]. This has caused disadvantageous impacts on the fundamental processes of plants growth and production and finally bringing great damage to global agriculture and food security [3]. The improvement for crop yield-stability dealing with low phosphate stress has been partially accomplished via human-selection breeding through natural genetic variations [3,5]. Further, genetic engineering of plants for higher phosphate-uptake efficiency may have far-reaching effects to improve phosphate starvation tolerance. The public availability of genetic variations (e.g., 3010 diverse Asian culture rice) provides an invaluable resource for rice genomic research and breeding [68]. To examine the associations of genetic variations with agronomic traits (not only root system traits) under low phosphate conditions is of paramount importance to guide and accelerate rice breeding of sustainable agriculture.
